# Intensive Care Unit Risk Assessment: A Systematic Review

**DOI:** 10.18502/ijph.v49i8.3865

**Published:** 2020-08

**Authors:** Abbas HOMAUNI, Sanaz ZARGAR BALAYE JAME, Ebrahim HAZRATI, Nader MARKAZI-MOGHADDAM

**Affiliations:** 1. Department of Health Economics and Management, School of Public Health, Tehran University of Medical Sciences, Tehran, Iran; 2. Department of Health Management and Economics, School of Medicine, AJA University of Medical Sciences, Tehran, Iran; 3. Department of Anesthesiology, AJA University of Medical Sciences, Tehran, Iran

**Keywords:** Intensive care unit (ICU), Failure mode and effect analysis, Risk assessment

## Abstract

**Background:**

It is of paramount importance to reduce the probability of clinical risks to improve the quality of health care services, make the relationship between service providers and patients more effective, enhance patient satisfaction, and decrease the rate of complaints regarding medical errors in hospitals. This study aimed at detecting potential and unacceptable risks occurring in the hospital ICUs.

**Methods:**

In this systematic review, all studies examining the risk assessment of ICUs in hospitals using Failure Mode and Effect Analysis method were reviewed. Google scholar, PubMed, Scopus, SID, Magiran and Web of Science databases were searched to find relevant articles published from 1980 to 2019.

**Results:**

The most frequent failures detected in the reviewed articles consisted of high risk of infection inwards for medical and nursing operations, high infection rates inwards for medical devices’ operation within the unit, and early discharge. Moreover, the processes through which potential high-risk Failures were examined in these studies were injection or prescription process, suction process, the process of inserting or removing endotracheal tubes, the process of transferring patients from the operation room to the unit or vice versa, pressure ulcers, and processes related to the medical devices’ operation.

**Conclusion:**

There are many possible reasons for failure occurring throughout these processes, and the failure modes occurring in these processes are more probable to cause serious damages to patients, have high repeatability with low probability of failure detection as the failures cannot be discovered by the personnel.

## Introduction

People are aware of and in agreement regarding the significance and necessity of health. In other words, health is a capability that values human life. It is a kind of wealth to human beings, and hospitals are one of the important organizations playing a critical role in this regard (1). Hospitals are one of the main social organizations and play a vital role in improving the health status of a country and providing health services to society (2). Due to individuals’ changing health status and the rise of chronic diseases, one of the hospital wards received particular attention over recent years is the ICU.

The intensive care unit (ICU) was first used in a polio epidemic in Copenhagen, Denmark. In this case, the physicians managed to reduce the mortality rate among polio from 90% to about 40% by respiratory assistance. The unit then evolved and become as it is known today (3). The ICU is considered to be the heart of the hospital; however, it needs more attention due to its special conditions such as medical devices and specialized work (4). The ICU beds are one of the most important and valuable resources of the healthcare system, which have dramatically reduced mortality rate over recent years (5). Human error is one of the problems common in all clinical sectors of a hospital, particularly the ICU.

One of the major issues in healthcare organizations is the quality of healthcare services. In this regard, one of the major components in hospitals is patient safety (6). Patient safety is a fundamental issue in hospitals, which falls within the scope of clinical risk management (7).

Patient safety is so important that in the latest edition of National Hospitals Accreditation standards emphasizes that hospitals are required to prioritize patient safety in their strategic goals and values and one of the main issues in patient safety in hospitals is medical error reporting, Rout Cause analysis of errors and identifying potential error and preventing them (8).

Human error in is inevitable during complex processes and measures, and one of the hospital wards with complex processes and measures is the ICU so that some human errors might be observed in this unit (9). More than 1,300,000 persons admitted to the intensive care unit in US hospitals have been injured due to unintentional incidents, causing many problems for patients and their companions (10). One of the main problems with the errors is the imposition of heavy costs on the health system, patients, and their families (11).

It seems to be of paramount importance to reduce the probability of clinical risks to improve the quality of health care services, make the relationship between service providers and patients more effective, enhance patient satisfaction, and decrease the rate of complaints regarding medical errors in hospitals (6). One of the methods to reduce clinical risks is to assess the probability of clinical risks in hospital wards.

Risk assessment is a logical method to quantitatively and qualitatively measure the risks and the potential consequences of potential incidents for individuals, materials, medical devices, and the environment, through which the effectiveness and efficiency of existing control methods can be investigated. Risk assessment can also provide valuable information in deciding to reduce risks, improve existing control systems, and plan for appropriate responses (12). Failure Mode and Effect Analysis (FMEA) is one of the most common risk assessment methods (13).

The FMEA method was first used in industry; however, the method, with the advent of a systemic approach to medical errors and the introduction of prospective approaches, was later applied in the field of treatment and health as such the JCAHO (2001) declared the regular implementation of FMEA in the intensive care unit as a prerequisite for all hospitals (14). The FMEA is a systematic, prospective, preventive, and team-based approach (15). The approach is to hinder an error or incident through defining, identifying, evaluating, preventing, or controlling the modes and effects of possible errors in a system or process before the end product or service is delivered to the customer. It also requires to predict errors and shows how to prevent them (11).

Totally, risk assessment can be done in different parts of the hospital. In a study, of the 6 selected emergency department processes, 51 potential error were identified, of which 16 were unacceptable (16).

Given the sensitivity of ICUs in providing healthcare services to patients and since the errors in this unit could lead to irreparable complications for patients, the aim of this study was detecting potential and unacceptable risks occurring in the hospital ICUs based on resent researches and the main question is what are the main risks identified in the Intensive Care Unit.

## Methods

### Ethical Approval

Ethical approval for this study was obtained from the research ethics committee of AJA University of Medical Sciences. Informed consent was obtained from all participants before the study.

This research was a systematic study aimed at identifying potential and unacceptable risks occurring in the ICUs of different hospitals. To this end, all studies conducted in Iran and other countries examining the risk assessment of ICUs in hospitals using FMEA method were considered.

Google scholar, PubMed, Scopus, SID, Magiran and Web of science databases were searched to find relevant articles published from 1980 to 2019.

There were several inclusion criteria. First, the article is on risk assessment. Second, the risk assessment is conducted using the FMEA approach. Third, the studied ward is one of the intensive care units (ICU, NICU, PICU, ICU-OH, and ICU). Articles not meeting the inclusion criteria were excluded from the study. To extract data from each article, tables listing potential Failures detected in the ICUs were considered so that the Failures with an RPN> 100 or Failures with an unacceptable likelihood were found.

PubMed, Web of Science, and Scopus databases were searched using keywords such as Proximate causes OR Prospective risk assessment [OR/Title] OR RPN [Title/Abstract]) OR risk priority number [Title/Abstract]) OR RCA [Title/Abstract]) OR Root cause analysis [Title/Abstract]) OR Retrospective risk assessment [Title/Abstract]) OR Clinical hazard analysis [Title/Abstract]) OR Hazard analysis [Title/Hazard assessment [Title/Abstract]) OR hazard management [Title/Abstract]) OR hazard assessment [Title/Abstract]) OR risk management [Title/Abstract]) OR patient safety management [Title/Abstract]) OR patient safety [Title/Abstract]) OR safety management [Title/Abstract]) OR Process failure mode effect analysis [Title/Abstract]) OR PFMEA [Title/Abstract]) OR healthcare failure mode effect analysis [Title/Abstract]) OR HFMEA [Title/Abstract]) OR FMEA [Title/Abstract]) OR failure mode effect analysis [Title/Abstract]) AND (((critical care medicine [Title/Abstract]) OR ICU [Title/Abstract]) OR Intensive care unit [Title/Abstract]) OR intensive care medicine [Title/Abstract]). The same search strategies were used for the three databases. Farsi search was also performed using keywords such as risk assessment, intensive care unit (children, infants, burn intensive care, open heart intensive care), and FMEA.

### Detection of Papers

After surfing the aforementioned databases using the keywords, 2593 articles were found, of which 120 articles were repeated in different databases and excluded from the study, and 2473 papers were concerned.

### Evaluation of Papers

After studying the article titles, 2348 articles were removed since they were not relevant to the research objectives, and 125 articles were selected to be studied. Afterward, after studying the abstracts, 81 articles were also removed due to the same reason, and 44 full-text articles were studied for final selection.

### Selection of Papers

In the last step, 26 articles out of 44 full-text articles were excluded from the study (for example, articles assessing risks using a method but FMEA, or articles assessing risks in a ward other than ICU). Finally, 18 articles were selected for final analysis.

**Fig. 1:**
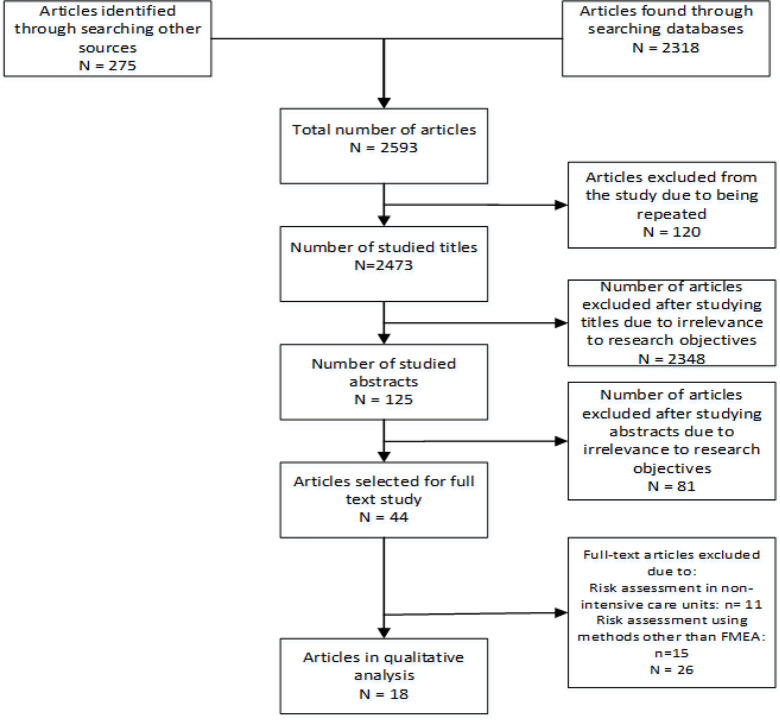
Article search process in the present systematic review

The search process was carried out by two individuals in order to ensure the accuracy of the search strategy and the obtained results, resulting in their final confirmation. Figure 1 represents the search strategy of the databases.

As the main limitation of the study, there was no access to some full-text articles that seemed to meet the research objectives. We could reach two full-text articles by contacting their authors.

## Results

Eighteen articles were selected for final review, of which 14 papers were in English and four papers were in Farsi. For the articles whose RPN numbers were selected from 1 to 5, the final numbers were multiplied by 8 for the sake of homogeneity. Table 1 illustrates the type of intensive care unit involved in the reviewed articles on risk assessment using the FMEA method. Table 2 shows the Failure modes with the highest RPN in the reviewed articles.

**Table 1:** Type of intensive care units in the reviewed articles

***No.***	***Type of intensive care units***	***Frequency***	***%***
1	ICU	14	77.8
2	PICU	2	11
3	NICU	1	5.6
4	Burn Intensive Care	1	5.6
Total		18	100

**Table 2:** Failure modes with the highest RPN

***No.***	***Process or Activity***	***Failure mode***	***RPN***
1	-	High infection rate inwards for medical and nursing operations	1000
2	-	High infection rate inwards for medical devices’ operations	1000
3	-	Early discharge	1000
	Prevention of venous thromboembolism in critically ill patients	Failure to maintain the performed evaluations and to detect complications	810
5	Prevention of venous thromboembolism in critically ill patients	Lack of a protocol for the maintenance of measurements and evaluations and the detection of complications	810
6	-	Endotracheal tube obstruction	800
7	Process of prescribing and taking medication in burn intensive care unit	Likelihood of Failures when being checked for dangerous medication by second nurse or in the case of incorrect confirmation of medication (from prescription category)	800
8	Prevention of venous thromboembolism in critically ill patients	No prescription for evaluations and measures	729
9	General process: Endotracheal tube (ETT) suction	Inaccurate evaluation regarding the necessity of suctioning	648
10	Process of prescribing and taking medication in burn intensive care unit	Not checking the route of administration, drug name, drug dosage, route of injection to patients (from medication use category)	640
11	-	Medication Failures caused by the physician’s inattention in managing patient treatment and providing oral instructions	640
12	Kidney transplant treatment	early circuit clotting	640
13		Failure to respond to alarms from patient-connected medical devices because of ignorance	630
14	-	Failure to respond to alarms from patient-connected medical devices because of not hearing the alarm (when the personnel are out of the ward)	630
15	-	Failure to respond to alarms from patient-connected medical devices because of not hearing the alarm (when the patient is far away or when the isolation room is closed)	630
16	-	Failure to respond to alarms from patient-connected medical devices because of high work load and delay in response	630
17	General process: Endotracheal tube (ETT) suction	Aseptic technique with sterilized gloves (improper technique)	576
18	Kidney transplant treatment	Real dosage lower than the prescribed one	576
19	-	Mistakes in prescribing kind and dosage of medication, caused by physician’s illegible handwriting	560
20	-	Medication Failures caused by nurses’ negligence	560

Table 2 lists 20 Failure modes with the highest RPN in the reviewed articles. The highest RPN (RPN=1000) was obtained for high infection rates in the intensive care unit for medical, nursing, and medical devices’ operations as well as the early discharge of patients.

In Table 3, the most frequent processes indicating unacceptable Failure modes in different articles are presented.

**Table 3:** Frequency of articles reviewing processes with unacceptable Failure modes

***No.***	***Process or Activity***	***Number of articles in which the process had unacceptable failure mode***
1	injection or drug administration	10
2	Suction	6
3	inserting or removing the endotracheal tube	6
4	transferring the patient from operation room to ward or vice versa	4
5	Pressure ulcer	3
6	Operations of medical devices	3

To obtain RPN number, FMEA team is asked to score failure modes based on severity (if error occurs, how much the patient is affected), probability of occurrence and probability of error detection from 1 (minimum severity, minimum occurrence, and maximum probability of error detecting) to 10 (maximum severity, maximum occurrence, and minimum probability of error detecting). If the result of multiplying these 3 numbers is higher than 100, then failure mode is unacceptable.

The above table shows number of articles in which processes mentioned had unacceptable failure mode. For example, 10 reviewed articles found the Failures of drug injection processes to be unacceptable Failures (RPN>100). Other processes undertaken in the ICUs, which were regarded as unacceptable Failures, were suction process, the process of inserting or removing endotracheal tubes, the process of transferring patients from the operation room to the unit or vice versa, pressure ulcers, and processes related to the medical devices’ operations.

## Discussion

Considering the inclusion criteria and reviewing the selected articles, 18 articles were selected for the final analysis. The selected articles assessed risks using the FMEA method in the intensive care units (NICU, PICU, ICU, and burn intensive care unit). According to Table 1, a majority of the articles (14 articles) examined risk assessment in the ICU.

In the reviewed articles, the highest RPN score (RPN=1000) of the potential Failure modes was obtained for high infection rate in the wards for intra-ward operations, the wards for medical and nursing operations, as well as early discharge (17). Surveys show that 5%–25% of patients referring to hospitals are infected with nosocomial infections, with frequencies of 25% in developed countries and 50% in developing countries. The rate of nosocomial infection in the intensive care unit is 5 to 10 times as high as the other units, leading to numerous problems such as mortality and enhanced medical costs (18). The smallest mistake in the ICU would infect patients and cause irreparable damages. This issue is well illustrated in the present article.

Other Failure modes with high RPN scores in the concerned articles were failure to maintain the undertaken evaluations and to detect complications (RPN=810) and lack of a protocol for the maintenance of measurements and evaluations and the detection of complications (RPN=810) (19). Another Failure with a high RPN score (RPN=729) was lack of a protocol for the maintenance of measurements and evaluations regarding the pulmonary thromboembolism in ICU patients. All the three Failures were related to the process of preventing pulmonary thromboembolism in critically ill patients (17). Thromboembolism is one of the complications which occur frequently and unnoticeably in the intensive care units, enhancing the mortality rate among the patients (20, 21). The pulmonary thromboembolism prevention is a critical process, and that mortality rates would be significantly decreased through identifying potential Failures in this process.

The endotracheal tube obstruction during the endotracheal tube insertion process was reported as a dangerous and unacceptable Failure with RPN= 810 (22). In general, Failures modes associated with endotracheal tube insertion were considered as unacceptable (11, 23–26), proposing that the endotracheal intubation process is one of the fundamental processes in the intensive care units and that the smallest Failure could cause irreparable damages to the patients. As a result, a detailed examination of the processes involved in inserting or removing the endotracheal tube would contribute to the detection of potential Failures to be removed to the extent possible.

In the reviewed articles, the other prevalent Failures among the Failures with high RPN scores were observed during the process of drug administration, injection, or medication use in the intensive care unit (11, 17, 23, 24, 27–34). Medication error refers to as a preventable incident that would lead to improper medication use, and ultimately to a patient’s damage or even death (35). In the intensive care units, the likelihood of this type of Failures as well as the patients’ mortality rate is enhanced due to the complex occupational conditions of the personnel, including physicians and nurses, as well as the large volume of medications used in these units (36). Moreover, given that patients in the intensive care units are unconscious, they cannot report side effects of the medication errors; hence, medication errors are less detected and the smallest errors regarding the medication dosage or medication administration time would lead to irreparable damages to the patients or even their death (37). One of the most frequent processes in intensive care unit, in which the probability of Failure is extremely high and Failures could impose high life and financial costs are related to prescribing, injecting, and taking medication in this ward. Much more attention should be paid to such Failures, and potential Failures need to be detected and prevented.

One more potential Failure with a high RPN score in the reviewed articles was reported for the operation of medical devices or their malfunctioning or personnel’s inattention to the alarms, which would lead to some damages to patients admitted to the intensive care units (17, 24, 38). A majority of Failures in the ICUs are due to the lack of attention to alarms and warnings from medical devices (17). In addition, medical devices’ failures in special units or their malfunctioning could expose patients to many risks; thus, regular and periodic monitoring and repair of medical devices by the hospital’s medical devices unit is recommended. Moreover, to reduce human errors, some strategies are needed to increase the personnel’ sensitivity to the alarms sent from the medical devices.

Another process in the reviewed articles with RPN>100 is the process of transferring the patient to the intensive care unit or vice versa (11, 23, 39). One of the care challenges in intensive care units is patient safety because these patients are exposed to a variety of complications and injuries due to their special health status. One of the measures threaten patient safety in this unit is transferring the patient from ICU to other wards for diagnostic and therapeutic operations and vice versa. Complications that could endanger the patients in the intensive care units include decreased or increased blood pressure, arrhythmia, altered respiratory rate, decreased arterial oxygenation, and medical devices’ failure. In this regard, the prevalence of problems posed by transferring patients to the ICUs and vice versa varies from 6 to 70% in different studies (40). Regarding the patients’ health status in these units, the transfer of patients to the intensive care units or vice versa must be undertaken carefully. The smallest Failures would make the patient experience irreparable damages. To examine the process of transferring the patient to the ICUs, the potential Failures should be detected to greatly reduce the damage to the patients.

Hospitalized patients are prone to bedsore because of inactivity and sleeping on beds. In the reviewed articles, Failures during the care processes to prevent bedsore were unacceptable Failures with RPNs >100 (11, 24, 38). Pressure ulcers are caused by ischemic pressure injuries to different body organs. According to the reviewed articles, patients admitted to intensive care units are at higher risk of bedsore than patients admitted to other hospital wards as they are hospitalized and inactive for a longer time and the disease severity is higher as these patients usually receive high APPACHE scores (41). Bedsore should be of concern because this problem generally affects many patients, their families, health centers, and even society through imposing physical, financial, and social consequences (42). High costs are annually paid for preventing and treating pressure ulcers at care centers around the world. In the United Kingdom, for example, 3.2% of total health care costs (equivalent to $ 680 million from the NHS annual budget) is to prevent and treat pressure ulcers (42). Obviously, the prevention of bedsore is critical for patients admitted to the intensive care units because the patients are prone to severe injuries and even death if no preventive measure is adopted and since it is costly to the health system.

## Conclusion

Critical and recurrent processes and Failure modes in ICUs were detected based on previous research. This issue is of paramount significance because the careful examination of these processes and the detection of potential Failures would facilitate the adoption of effective measures to prevent the Failures.

The main processes in the intensive care units which pose potentially high risk of Failures are the process of medication injection or administration, suctioning, the process of inserting or removing endotracheal tube, the process of transferring the patient from operation room to the ward or vice versa, bedsore, and the processes related to the operation of medical devices. Failures are likely to occur during these processes for a variety of reasons, and the Failure modes occurring during these processes are highly likely to cause severe injuries to the patients and have high repeatability with low probability of Failure detection. In addition to studying the various processes in the intensive care units, these processes should be carefully carried out, and necessary training and control mechanisms need to be applied to prevent any potential human Failure during such processes. Increasing the life expectancy and the quality of life among the patients admitted to this unit would greatly avoid the high costs of Failures in this ward.

## Ethical considerations

Ethical issues (Including plagiarism, informed consent, misconduct, data fabrication and/or falsification, double publication and/or submission, redundancy, etc.) have been completely observed by the authors.
